# Prevalence and Predictors of Driving after Prescription Opioid Use in an Adult ED Sample

**DOI:** 10.5811/westjem.2020.3.44844

**Published:** 2020-06-19

**Authors:** Aaron D. Dora-Laskey, Jason E. Goldstick, Brooke J. Arterberry, Suni Jo Roberts, Rebecca L. Haffajee, Amy S.B. Bohnert, Rebecca M. Cunningham, Patrick M. Carter

**Affiliations:** * University of Michigan, Department of Emergency Medicine, Ann Arbor, Michigan; † University of Michigan, Department of Psychiatry, Ann Arbor, Michigan; ‡ University of Michigan Addiction Center, Ann Arbor, Michigan; § University of Michigan Injury Prevention Center, Ann Arbor, Michigan; ¶ Iowa State University, Department of Psychology, Ames, Iowa; || University of Michigan School of Public Health, Department of Health Management and Policy, Ann Arbor, Michigan and RAND Corporation, Boston, Massachusetts; # University of Michigan, Department of Anesthesiology, Ann Arbor, Michigan; ** VA Center for Clinical Management and Research, VA Ann Arbor Healthcare System, Ann Arbor, Michigan; †† Hurley Medical Center, Department of Emergency Medicine, Flint, Michigan

## Abstract

**Introduction:**

Prescription opioid use and driving is a public health concern given the risks associated with drugged driving, but the issue remains under-studied. We examined the prevalence and correlates of driving after taking prescription opioids (DAPO) among adults seeking emergency department (ED) treatment.

**Methods:**

Participants (aged 25–60) seeking ED care at a Level I trauma center completed a computerized survey. Validated instruments measured prescription opioid use, driving behaviors, and risky driving. Patients who reported past three-month prescription opioid use and drove at least twice weekly were administered an extended study survey measuring DAPO, depression, pain, and substance use.

**Results:**

Among participants completing the screening survey (n = 756; mean age = 42.8 [standard deviation {SD} =10.4]), 37.8% reported past three-month prescription opioid use (30.8% of whom used daily), and 14.7% reported past three-month DAPO. Of screened participants, 22.5% (n = 170) were eligible for the extended study survey. Unadjusted analyses demonstrated that participants reporting DAPO were more likely to use opioids daily (51.1% vs 15.9%) and had higher rates of opioid misuse (mean Current Opioid Misuse Measure score 3.4 [SD = 3.8] vs 1.1 [SD = 2.1]) chronic pain (80.7% vs 42.7%), and driving after marijuana or alcohol use (mean intoxicated driving score 2.1 [SD = 1.3] vs 0.3 [SD = 0.8]) compared to patients not reporting DAPO (all p<0.001). Adjusting for age, gender, employment, and insurance in a logistic regression model, participants reporting DAPO were more likely to report a chronic pain diagnosis (odds ratio [OR] = 3.77, 95% confidence interval [CI], 1.55–9.17), daily opioid use (OR = 3.81, 95% CI, 1.64–8.85), and higher levels of intoxicated driving (OR = 1.62, 95% CI, 1.07–2.45). Alcohol and marijuana use, depression, and opioid misuse were not associated with DAPO in adjusted analyses.

**Conclusion:**

Nearly one in six adult patients seeking ED care reported DAPO. The ED may be an important site for interventions addressing opioid-related drugged driving.

## INTRODUCTION

Motor vehicle collisions (MVC) are a leading cause of death in the United States (US) (37,133 roadway fatalities in 2017),^1^ and are estimated to cost more than $41 billion annually.^2^ Since the increases in opioid pain reliever prescribing throughout the 1990s,^3^ the proportion of fatally injured drivers testing positive for prescription opioids has increased sevenfold.^4^ Despite improved prescribing practices in response to the ongoing opioid overdose crisis, US opioid prescribing rates remain threefold higher than they were in 1999.^5^ This highlights the need for more research into driving under the influence of opioids, including determining the rates and correlates of contemporaneous driving and opioid use. Such data would better inform road safety efforts in this area, which lag behind those addressing alcohol-impaired driving (e.g., developing roadside screening assays).^6^

Opioids are associated with a dose-dependent diminution in motor and sensory function in human studies,^7^ where experiments in healthy volunteers have demonstrated deleterious effects of opioids on the neurocognitive and psychomotor functions requisite for safe motor vehicle operation (e.g., logical reasoning, reaction time, eye-hand coordination).^8^ While older studies of driving behavior among patients on chronic, stable. opioid regimens largely affirmed the notion that opioid medications did not dynamically impair driving ability,^9^–^12^ few of these studies measured real-world driving, and may not have accounted for the entire breadth of cognitive and motor skills necessary to drive safely.^13^ Further, the relevance of prior research supporting the safety of driving after taking prescription opioids (DAPO) has been limited by a focus on patients being treated for cancer or opioid use disorders,^14^,^15^ reliance on historical controls, small samples of opioid users,^16^–^18^ and methodological concerns (e.g., lack of blinding, or using purely psychological tests to estimate driving aptitude).^14^,^19^ Indeed, as noted in a review by Gjerde et al,^20^ unlike prior epidemiological studies, those performed after 1998 in most cases (17 of 25 identified) *did* identify a significant association between opioid use and MVC risk.

Given the dramatic increase in the rates of opioid prescribing to treat chronic non-cancer pain^21^ and the prevalence of opioid use disorders,^22^ there is a need to revisit the risks associated with DAPO. More recent epidemiological studies have cast doubt on the safety of driving after taking prescription opioids, linking both MVC risk to the initiation of prescription opioids,^23^,^24^ and higher prescribed opioid dosages to road traffic injuries.^25^ Further, while co-occurring use with alcohol and other drugs (e.g., cannabis) is common among fatally injured drivers testing positive for opioid analgesics,^4^ there are few published data on the relationships between DAPO, other drugged driving, and driving under the influence of alcohol (DUI) in ambulatory samples.

Emergency providers must be equipped to provide informed advice to patients using opioids about the safety of such tasks as motor vehicle operation, and are frequently charged with assessing patients for the presence of risky opioid use. While emergency physicians (EP) are responsible for only 4% of all opioid prescriptions written annually in the US, approximately 20% of all emergency department (ED) prescriptions are for opioid analgesics.^26^ Because persons with chronic non-cancer pain and other painful conditions often require urgent or unscheduled care,^27^–^29^ the ED may be an opportune site for studying rates and correlates of DAPO. Understanding the risks of DAPO may better allow ED care providers to present informed advice to patients about the safety of prescribed opioid analgesics, and better equip them to screen patients for risky opioid use and driving behaviors. However, few studies on driving behaviors among patients taking prescription opioids have been conducted in this setting. Greater understanding regarding the prevalence of DAPO among ED populations—and its associated predictors—could advance the ability of emergency care providers to screen for dangerous opioid use, provide counseling and/or interventions to reduce DAPO, and assess the need for treatment referral in patients with suspected opioid use disorders.

**Population Health Research Capsule**

What do we already know about this issue?

*Drugged driving crashes are a serious public health concern, but the relationship between driving and prescription opioid use is poorly understood*.

What was the research question?

What is the prevalence of adult ED patients driving after prescription opioid use?

What was the major finding of the study?

*Nearly 1 in 6 adult ED patients reported driving after taking prescription opioids in the past 3 months*.

How does this improve population health?

*Understanding the prevalence and risks of prescription opioid drugged driving could help emergency physicians better identify high-risk patients for interventions*.

In this study, we determined the prevalence of prescription opioid use and driving after prescription opioid behavior (and their demographic correlates) in a screening sample of adults ages 25–60 seeking ED care, then examined the predictors of DAPO in the subset of patients who both reported prescription opioid use and drove regularly (at least twice per week). We hypothesized that adult ED patients who reported DAPO would be more likely to misuse these prescription opioids, have associated mental health and substance use problems, and engage in other risky driving (including driving after alcohol or marijuana use).

## METHODS

### Study Design

We conducted a cross-sectional analysis of prescription opioid use and driving behaviors among adult patients seeking emergency department care as part of the Health Behaviors and Prescription Opioids Study (HBPOS). The University of Michigan Institutional Review Board approved the study protocol, and a Certificate of Confidentiality was obtained from the National Institutes of Health.

### Study Setting and Population

Patients were recruited from the University of Michigan Health System ED, a Level I trauma center located in Washtenaw County (median household income $62,484, 74.4% white), with an annual ED patient census of ~85,000 adult patients.^30^

### Study Protocol

Study participants were recruited seven days a week (excluding holidays) between September 22, 2016–February 1, 2017, by trained research assistants (RA), between the hours of 9 am and 10 pm. Potentially eligible participants were identified using electronic patient tracking logs, and approached for screening in private treatment rooms. Because both adolescents/young adults and elderly patients may differ in their risky driving behaviors, the sample was limited to adults aged 25–60.

We excluded patients from screening if they were cognitively impaired by intoxication, illness, or injury; lacked adequate command of English; were in police or corrections custody; were presenting for evaluation and treatment of sexual assault or suicidal ideation; were classified by ED staff as a Level I trauma; or required special precautions due to the risk of infectious disease exposure. Patients reporting a history of schizophrenia were excluded from the survey due to both a concern about their ability to provide adequate informed consent and the degree of psychosocial needs that such patients often require during their ED visit, which precludes adequate time for completion of all study procedures. When there was concern with the capacity of patients with other psychiatric diagnoses (e.g., depression or bipolar disorder) or cognitive impairment to provide informed consent, the RA administered a Mini-Mental Status Exam.

RAs obtained verbal informed consent for the screening survey, which was completed by the participant on a computerized tablet. Participants were remunerated with a gift worth ~$1.00 (e.g., Sudoku booklets). Patients completing the screening survey were eligible for the extended survey if they reported any past three-month prescription opioid use, and drove at least twice per week in the prior three months. Survey participants completing the extended survey provided written consent. All surveys were administered privately (i.e., family/friends were not allowed to see questions) using tablet computers, and paused as required for medical care. Participants were remunerated $20 cash for completing the extended survey. All patients were given a community resource brochure with local mental health and substance use resources.

### Measurements

#### Driving After Prescription Opioids (DAPO)

The main outcome variable, driving after prescription opioids (DAPO), was determined by any affirmative response to the question, “In the past three months, how many times did you drive after taking opioid pain medications?”

#### Demographics

We obtained sociodemographics (eg, age, gender, race, employment/school status, disability, and insurance coverage) using self-report measures. Race was dichotomized as White vs non-White for analysis. Employed/school was coded as positive for participants reporting full-time or part-time employment or being a student when queried about current employment status; disability was determined by the selection of the response “Unemployed, disabled” within the same measure. Private insurance included positive responses to either having private insurance (yes/no) or group insurance (yes/no).

#### Opioid Use

We defined daily opioid use as a response of “Daily or almost daily” to the question, “In the past three months, how often have you used opioid pain medications (For example: Vicodin, Codeine, OxyContin, morphine, oxycodone, hydrocodone, methadone, hydromorphone, meperidine, fentanyl, or Norco)?” Opioid misuse behaviors were measured by the sum of eight items from the Current Opioid Misuse Measure (COMM), a validated scale.^31^

#### Risky Driving Behaviors and Consequences

For the purposes of this study, we constructed composite risky driving (eg, speeding, tailgating) and intoxicated driving (alcohol and marijuana) scores by summing the responses of 16- and 7-question subsets, respectively, of the Risky Driving Survey.^32^ Responses were on a five-point Likert scale (1-Never, 2-Rarely, 3-Sometimes, 4-Often, 5-Always). Driving under the influence of opioids was determined by an affirmative answer to the question, “In the past 30 days, how often have you driven while you were feeling the effects of opioid pain medications, either alone or with alcohol, other drugs, and/or medications?” Patients’ plans to drive after prescription opioids in the next three months were measured on a 10-point Likert scale (from “Not very likely” to “Very likely), and were dichotomized as either “Less likely” (≤5) or “More likely” (≥6).

#### Depression and Chronic Pain

We determined depression severity using the Patient Health Questionnaire-9 (PHQ-9),^33^ a 27-point scale where higher scores indicate greater frequency of depressive symptoms. Chronic pain was assessed with the question, “Have you been told by a doctor that you have chronic pain (Yes/No)?”

#### Alcohol and Marijuana Use

We measured alcohol and marijuana use by summing numerically coded responses to the National Institute on Drug Abuse and the Alcohol, Smoking and Substance Use Involvement Screening Tests (NIDA-ASSIST).^34^,^35^

### Data Analysis

We performed statistical analyses using SAS 9.4 (SAS Institute, Cary, NC). First, we examined data from the screening survey to determine the prevalence of DAPO among the general adult ED population, and calculated descriptive statistics for this sample. Second, we examined DAPO among the subset of screening participants who received the extended study survey (ie, those who reported past three-month opioid use and twice-weekly driving). We limited the analysis of factors associated with DAPO to those taking the extended study survey because many of the measures of interest (e.g., high-risk driving behavior, substance use) were only measured in that subsample. We conducted bivariate comparisons between those who did and did not endorse DAPO among respondents to the extended study survey using *t*-tests for continuous variables and χ^2^ tests for categorical data. Third, adjusted comparisons between participants with and without DAPO were modeled using logistic regression. We added variables to the logistic regression model sequentially, beginning first with demographics, and then substance use, depression, and chronic pain; and, finally, driving behaviors. The determination of predictor variables in the adjusted analysis was based on both theoretical considerations and parsimony (given the relatively small study sample).

## RESULTS

### Rate of Driving After Prescription Opioids Among the Screening Sample of Adult Patients Seeking Emergency Treatment

The recruitment flowchart is shown in Figure. A total of 1111 ED patients ages 25–60 were approached; 756 (68.0%) of these patients completed the screening survey, of whom 170 (22.5%) were eligible and agreed to be enrolled in the extended study survey, providing complete data on key variables.

Overall, the screening sample (n = 756) had a mean age of 42.8 (standard deviation [SD] = 10.4), was 61.4% female, 74.5% White, and 25.1% low income (<$20,000/year). Among screened participants, 37.8% reported past three-month prescription opioid use (30.8% of whom reported daily use) and 14.7% reported past three-month DAPO. Among those reporting driving after taking opioids, 53.2% reported that they had also been driving under the influence of opioids, and 35.1% reported that they planned to continue driving after taking prescription opioids in the subsequent six months.

### Extended Study Survey Analysis

Among screened participants, 22.5% (n = 170) met the study criteria of past three-month prescription opioid use and regular driving (i.e., at least twice weekly) and completed the extended study survey. The remainder of reported analyses are on this subsample of participants.

#### Unadjusted Analysis

The bivariate analysis of DAPO and its predictors is shown in Table 1. Participants reporting DAPO were more likely than those not reporting DAPO to use opioids daily (51.1% vs 15.9%), have higher levels of opioid misuse (mean COMM score 3.4 [SD = 3.8] vs 1.1 [SD = 2.1]), and have higher rates of chronic pain (80.7% vs 42.7%; all p<0.001). Further, participants endorsing DAPO demonstrated higher rates of other impaired driving behaviors (e.g., driving after marijuana or alcohol use): the mean intoxicated driving score for those reporting DAPO was 2.1 [SD = 1.3], compared with 0.3 [SD = 0.8]) among those not reporting DAPO.

#### Logistic Regression Model

Logistic regression results are shown in Table 2. Addition of the substance use, depression, and chronic pain variables substantially improved model fit (Model 2; p < 0.001) relative to the demographics-only model, and addition of the driving and opioid use characteristics substantially improved fit (Model 3; p < 0.001) relative to the second model. The final model (Model 3) had an area under the receiver operator characteristic curve of 0.82, indicating good model discrimination. Adjusting for age, gender, employment, and insurance, patients reporting DAPO were more likely to disclose a prior diagnosis of chronic pain (odds ratio [OR] = 3.77, [95% confidence interval {CI}, 1.55–9.17), daily opioid use (OR = 3.81, 95% CI, 1.64–8.85), and greater frequency of intoxicated driving (OR = 1.62, 95% CI, 1.07–2.45) compared to the non-DAPO group. Depression, alcohol use, marijuana use, and prescription opioid misuse were not associated with DAPO in the adjusted model, nor were there any significant associations with sociodemographic covariates.

## DISCUSSION

To our knowledge, this is the first study of DAPO prevalence and its relationship to other substance use and risky driving behaviors in an ED sample. Nearly one in six adults surveyed during the enrollment period reported DAPO during the prior three months. Among regular drivers, those who reported DAPO were more likely to also report driving after marijuana and alcohol use, highlighting this as a particularly high-risk sample of drivers. Over a third of those reporting DAPO reported future plans to drive after taking opioids, suggesting a substantial need for prevention efforts among this group.

Among our screening cohort of adults ages 25–60 seeking ED care, 37.8% of participants reported past three-month prescription opioid use (medical use or misuse). While direct comparisons between our study sample and other ED populations are difficult given the dearth of published data, this is in contrast to the 37.8% (note: coincidentally identical value) past 12-month prevalence of prescription opioids among respondents to the 2015 National Survey on Drug Use and Health; the latter includes only non-institutionalized, civilian adults, and may reflect a lower risk population than do ED samples.^36^ In light of the persistently elevated rates of both US opioid prescribing and opioid-related deaths^37^ – and considering prescription opioids’ abuse potential and associated overdose risk – the prevalence in this study illustrates the ongoing significance of prescription opioid use for emergency care providers. Further, because patients with complications from opioid use disorders frequently access EDs for care, the ED may be an ideal venue in which to provide interventions aimed at reducing opioid-related harms (e.g., overdose).^38^

Our findings suggest that DAPO is prevalent among adult ED patients, with 14.7% of study participants reporting driving after prescription opioids. Further, a majority of those in the DAPO group also reported driving while under the effects of these drugs, and more than a third of these individuals planned on driving after taking opioids in the future. While we are not aware of any analogous published data on the prevalence of DAPO among adult ED patients in the same age range, a study of 586 emerging adults (ages 18–25) seeking ED care demonstrated that 24% of surveyed participants reported past 12-month drugged driving, 19% of whom reported DAPO.^39^ In the 2013–2014 National Roadside Survey, 7.5% of drivers (ages 16 years and older) reported past two-day use of prescription opioids.^40^ Our study was not sufficiently powered to reveal more definitive relationships between DAPO and MVC outcomes (only two crashes were reported in the extended study sample [data not shown]). However, considering epidemiological studies linking prescription opioid use and MVCs,^13^,^41^ and the prevalence of both past three-month prescription opioid use and future plans to drive after prescription opioid use in our study population, ED screening and interventions for risky opioid use and driving behaviors may have the potential to reduce opioid-related consequences such as MVCs.

We found that DAPO was more likely among participants who reported a prior diagnosis of chronic pain. This finding is consistent with prior studies examining the impact of opioid therapy in chronic pain patients, which have been associated with an increased risk of opioid use disorders,^42^ ED visits,^43^ overdose,^44^ and death.^45^ However, there are only sparse data on MVC risk among patients on prescribed chronic opioids. While several older studies of patients on opioid therapy for non-malignant pain (e.g., Galski et al, 2000)^17^ purported that stable doses of these drugs did not impair motor vehicle operation, such studies had key methodological limitations (discussed above). Chronic pain syndromes are prevalent in the US broadly,^46^ and among ED populations specifically,^28^ and are frequently treated with opioid pain relievers.^45^ Considering research linking prescription opioid use with cognitive impairments^9^ and impaired driving performance,^13^ our study highlights the importance of further research investigating chronic pain as a risk factor for opioid drugged driving and related morbidity.

The need for risk reduction around driving and opioids is underscored by our finding that drivers reporting DAPO were also more likely to drive under the influence of marijuana and/or alcohol. Emerging research is illuminating the relationships of marijuana and alcohol use and drugged driving behavior.^47^ In a study of younger adults, higher rates of opioid use correlated with higher frequencies of drugged driving; drugged driving, in turn, was associated with increased rates of hazardous drinking.^39^ Polysubstance use among drivers is an important public health problem. In a recently published study of 118 rural DUI offenders, 60% reported past-year drugged driving, and nearly half of those ever reporting drugged driving reported DAPO.^48^ In a 10-year analysis of Fatality Analysis Reporting System data published in 2017, 30% of fatally injured, opioid-positive drivers had blood alcohol levels ≥ 0.01 milligrams per deciliter.^4^ Our study findings, especially in the context of these data, suggest that DAPO may be a risk factor for other impaired driving behaviors, and further supports the importance of developing predictive tools for better identifying ED patients at risk for impaired driving.

This study suggests roles for primary and secondary prevention efforts in the ED aimed at reducing harms from prescription opioids.^49^ Considering the prevalence of DAPO in our sample, and epidemiologic data linking prescription opioids to increased MVC risk,^50^ ED prescribers may reasonably include these disclaimers when discussing the safety of DAPO, and consider such risks when deciding whether to initially prescribe an opioid analgesic. Studies have shown that opioid-alternative analgesics are as efficacious as opioids in treating acute extremity pain,^51^ and result in comparable pain control scores on post-discharge patient satisfaction surveys.^52^ ED prescribing guidelines may be effective at reducing the proportion of patients prescribed opioids on discharge,^53^ while electronic health record (EHR) default options for prescription opioids may affect emergency clinician quantity choice.^54^ Enhancing current EHRs with automatic reminders of the risks of DAPO may aid clinicians in providing this critical information to patients at the time they prescribe opioid medications.

EHR-integrated prescription drug monitoring program (PDMP) data may facilitate screening for potentially risky, prescription-opioid behaviors during the ED visit, and may be useful in assessing patients with daily use and/or chronic pain for potential interventions to reduce overdose risk.^55^ While recent ED-based research suggests that PDMP data may lack the predictive power to identify patients with opioid use disorders per se,^56^ previous studies have demonstrated that the availability of PDMP data may alter EP opioid prescribing.^57^,^58^ Further, states with more robust PDMPs have lower rates of opioid dispensing overall (including lower rates of dispensing high dosages) to patients on long-term, chronic opioid therapy, as well as lower rates of death from prescription opioid analgesics.^59^,^60^ Additional study is needed to better inform ED practitioners and patients alike about the risks of DAPO among patients with daily opioid use and/or chronic pain, and to develop optimal screening instruments and public health interventions to identify and address those who are at the greatest risk for DAPO-related harm.

## LIMITATIONS

Because our sample was recruited from a single, academic ED embedded in a city with high levels of education and income, results may not be generalizable to ED samples in dissimilar communities. Data were self-reported; however, previous research has supported the validity of these types of survey results.^62^ The exclusion of intoxicated patients, those in police custody, and the lack of RA coverage at night may have resulted in the underestimation of DAPO. Last, causal inferences are precluded by the cross-sectional nature of this study.

## CONCLUSION

Our results demonstrate that DAPO is prevalent among adult ED patients, is associated with other impaired driving behaviors, and that patients reporting DAPO are more likely to engage in risky driving behaviors in the future. These findings highlight the need to better understand other risks associated with DAPO, and to develop screening tools to better allow healthcare providers to identify at-risk individuals for potential interventions. Identifying predictive factors among routinely collected clinical data (e.g., past medical history and medications) may also help ED providers identify patients at risk for morbidity and mortality from drugged driving, especially when combined with data from PDMPs. Future research into the effects of prescription opioids on driving abilities and behaviors, including the role of dosing, frequency, and formulation, will be required to better understand the dynamic effects of these drugs on safe motor vehicle operation, and may better allow ED care providers to present informed advice to patients about the safety of prescribed opioid analgesics.

## Figures and Tables

**Figure:**
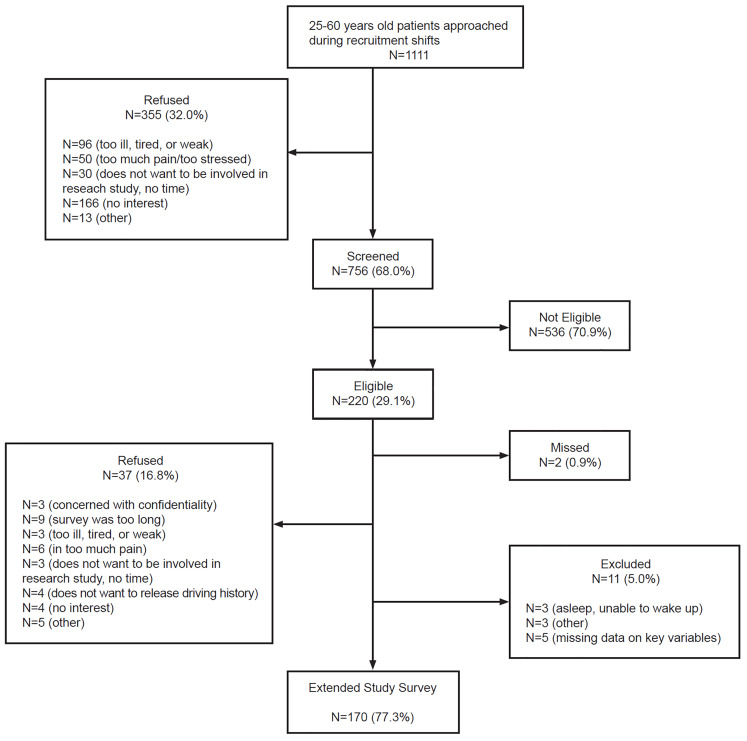
Health behaviors and prescription opioids study (HBPOS) recruitment flowchart (September 22, 2016–February 1, 2017).

**Table 1:** Bivariate analysis examining participants engaged in driving after taking prescription opioids (DAPO) compared with those not engaged in DAPO among the extended study sample (n = 170).

	DAPO (n = 88)	No DAPO (n = 82)	All (n = 170)
Sociodemographics			
Age†	43.1 (10.2)	42.6 (9.0)	42.8 (9.6)
Female gender‡	50 (56.8)	58 (70.7)	108 (63.5)
White race	71 (80.7)	17 (78.1)	135 (79.4)
Employed/in school*	42 (47.7)	55 (67.1)	97 (57.1)
Disabled	27 (30.7)	22 (26.8)	49 (28.8)
Private insurance*	41 (46.6)	52 (63.4)	93 (54.7)
Prescription opioid use			
Daily opioid use***	45 (51.1)	13 (15.9)	58 (34.1)
Total COMM score***	3.4 (3.8)	1.1 (2.1)	2.3 (3.3)
Risky driving/consequences			
3Intoxicated driving score***	2.1 (4.0)	0.3 (0.8)	1.25 (3.0)
1Risky driving score	6.7 (6.9)	5.1 (6.0)	5.9 (6.5)
Depression and chronic pain			
Total PHQ-9 score**	9.7 (5.9)	7.0 (5.3)	8.4 (5.8)
Chronic pain (n, %)***	72 (80.7)	35 (42.7)	106 (62.4)
Substance use			
Total ASSIST alcohol	5.4 (8.6)	4.4 (6.7)	4.9 (7.7)
Total ASSIST marijuana**	4.4 (1.7)	1.7 (3.9)	3.1 (5.9)

1 n_missing_=1

3 n_missing_=3.

† Continuous variables listed as mean, standard deviation.

‡ Categorical variables listed as n, %.

* p<0.05;

** p<0.01;

*** p<0.001.

*COMM*, Current Opioid Misuse Measure; *PHQ-9*, Patient Health Questionnaire-9; *ASSIST*, National Institute on Drug Abuse and the Alcohol, Smoking and Substance Use Involvement Screening Tests.

**Table 2:** Logistic regression models predicting driving after taking prescription opioids in the study population (n = 167*).

	Model 1 (OR, 95% CI)	Model 2 (OR, 95% CI)	Model 3 (OR, 95% CI)
AUC	0.65	0.77	0.82
−2 Log L	218.2	193.7	168.5
Chi-square (p < 0.05)		2 vs 1: p=0.00006 (DF=4)	3 vs. 2: p=0.00001 (DF=3)
Demographics			
Age	1.00 (0.96, 1.03)	1.01 (0.97, 1.06)	1.02 (0.97, 1.06)
Female gender	0.52 (0.26, 1.04)	0.54 (0.25, 1.18)	0.60 (0.25, 1.45)
Employed (full/part) or student	0.49 (0.25, 0.95)	0.89 (0.40, 1.94)	0.89 (0.38, 2.10)
Private insurance	0.60 (0.30, 1.17)	0.86 (0.41, 1.80)	1.10 (0.48, 2.53)
Depression, chronic pain, substance use			
PHQ-9 (total score)		1.07 (1.01, 1.15)	1.02 (0.95, 1.10)
Chronic pain		3.76 (1.70, 8.30)	3.77 (1.55, 9.17)
ASSIST alcohol (total score)		1.01 (0.95, 1.06)	0.98 (0.92, 1.05)
ASSIST marijuana (total score)		1.06 (0.99, 1.14)	0.99 (0.90, 1.08)
Opioid use and driving behaviors			
COMM score (total score)			1.09 (0.91, 1.31)
Daily opioid use			3.81 (1.64, 8.85)
Intoxicated driving (total score)			1.62 (1.07, 2.45)

* Three participants did not answer the intoxicated driving questions.

*OR*, odds ratio; *CI*, confidence interval; AUC, area under the curve; *PHQ-9*, Patient Health Questionnaire-9; *ASSIST*, National Institute on Drug Abuse and the Alcohol, Smoking and Substance Use Involvement Screening Tests; *COMM*, Current Opioid Misuse Measure.
